# Japanese Encephalitis Virus and Host Innate Immunity: Insights Into TLR Signaling, PRR Crosstalk, and Viral Immune Evasion

**DOI:** 10.1002/cbf.70259

**Published:** 2026-07-06

**Authors:** Mohammad Enamul Hoque Kayesh, Michinori Kohara, Kyoko Tsukiyama‐Kohara

**Affiliations:** ^1^ Department of Microbiology and Public Health, Faculty of Animal Science and Veterinary Medicine Patuakhali Science and Technology University Barishal Bangladesh; ^2^ Transboundary Animal Diseases Center, Joint Faculty of Veterinary Medicine Kagoshima University Kagoshima Japan; ^3^ Department of Microbiology and Cell Biology Tokyo Metropolitan Institute of Medical Science Tokyo Japan

**Keywords:** host–pathogen interaction, innate immunity, Japanese encephalitis virus, pattern‐recognition receptors, toll‐like receptors, viral immune evasion

## Abstract

Japanese encephalitis virus (JEV), a neurotropic flavivirus and a major cause of viral encephalitis, poses a significant global health threat due to its neuroinvasive potential. Host innate immune responses, particularly those mediated by pattern‐recognition receptors such as Toll‐like receptors (TLRs) and RIG‐I–like receptors, play critical roles in detecting JEV infection in neurons and glial cells, triggering antiviral defenses through induction of type I interferons (IFNs), inflammatory cytokines, and interferon‐stimulated genes. However, dysregulated inflammatory responses may contribute to neurodegeneration and disease severity. JEV has evolved multiple immune evasion strategies, including suppression of IFN signaling, modulation of host microRNAs, and exploitation of cellular pathways such as autophagy to facilitate viral replication and persistence. This review summarizes current knowledge regarding TLR and other PRRs‐mediated innate immune sensing during JEV infection, highlights the molecular mechanisms underlying viral immune evasion, and discusses the potential of TLR agonists as antiviral immunomodulators and vaccine adjuvants.

## Summary

Japanese encephalitis virus (JEV) remains a major cause of viral encephalitis worldwide, with limited therapeutic options available. Understanding host innate immune responses to JEV is therefore essential for developing improved antiviral and preventive strategies. This review summarizes how innate immune sensors, including TLRs and other pattern recognition receptors (PRRs), detect JEV in neural and glial cells and initiate antiviral defenses, while also contributing to inflammation‐associated neuronal injury. The review further highlights viral countermeasures that interfere with type I IFN signaling and host cellular pathways to facilitate viral replication and persistence. By integrating current findings, the review emphasizes the dual role of innate immunity in antiviral protection and immunopathology and discusses the potential of TLR‐targeted immunomodulators as therapeutic agents and vaccine adjuvants against JEV.

## 1 Introduction

Japanese encephalitis virus (JEV) is a leading cause of viral encephalitis worldwide, with an estimated 67,900 cases reported annually, highlighting its substantial public health burden despite ongoing vaccination efforts [1]. JEV is an emerging zoonotic, mosquito‐borne flavivirus endemic to tropical and subtropical regions of Asia and the Western Pacific, where more than 3 billion people are at risk of infection [2, 3]. Infection primarily causes acute neurological disease, particularly in children. Among patients who develop encephalitis, approximately 20%–30% die, while many survivors experience severe and long‐term neurological sequelae [4].

Taxonomically, JEV is a neurotropic member of the genus *Flavivirus* within the family *Flaviviridae*. Its genome consists of a single‐stranded, positive‐sense RNA of approximately 11 kb [5], encoding three structural proteins, including core (C), premembrane (prM/M), and envelope (E), and seven nonstructural proteins such as NS1, NS2A, NS2B, NS3, NS4A, NS4B, and NS5 [6]. Based on nucleotide sequence variation in the E gene, JEV is classified into five genetically distinct genotypes (GI–GV) [7].

JEV is maintained in an enzootic transmission cycle between vertebrate reservoirs and amplifying hosts, particularly water birds and pigs, together with culicine mosquitoes, especially *Culex* spp., which facilitate viral transmission and geographic spread [8, 9, 10]. Humans are considered incidental dead‐end hosts because they generally develop insufficient viremia to infect feeding mosquitoes.

Mosquito‐borne flaviviruses are increasingly expanding into new ecological niches worldwide, largely driven by environmental and climatic changes. Despite the widespread endemicity and continued geographic expansion of JEV, the molecular mechanisms underlying host–virus interactions remain incompletely understood, particularly those associated with innate immune sensing and downstream antiviral signaling pathways.

JEV infection induces the production of multiple cytokines and inflammatory mediators, including type I interferons (IFNs), tumor necrosis factor‐α (TNF‐α), and interferon‐gamma (IFN‐γ) [11]. The innate immune response constitutes the first line of defense against viral infection and plays a critical role in determining disease outcome by shaping subsequent adaptive immune responses [12]. Recent studies have further clarified how viruses engage innate immune signaling pathways, including Toll‐like receptor (TLR)‐mediated responses [13]. Understanding these interactions is important for elucidating JEV pathogenesis and identifying potential therapeutic and preventive strategies [14].

TLRs are pattern recognition receptors (PRRs) that detect conserved pathogen‐associated molecular patterns (PAMPs), including viral nucleic acids and proteins, and initiate signaling cascades that restrict viral replication and coordinate downstream immune responses [15]. Humans express 10 TLRs (TLR1–TLR10), whereas mice express 12 (TLR1–TLR9 and TLR11–TLR13) [16]. Cell surface TLRs, including TLR1, TLR2, TLR4, TLR5, TLR6, and TLR10 primarily recognize microbial and viral structural components, whereas intracellular TLRs, including TLR3, TLR7, TLR8, and TLR9, localize mainly to endosomal compartments and recognize viral RNA or DNA [17, 18, 19, 20, 21, 22]. Upon ligand recognition, most TLRs signal through the adapter protein MyD88, whereas TLR3 signals predominantly through TRIF, leading to activation of transcription factors that induce proinflammatory cytokines, chemokines, and type I IFNs [23, 24, 25, 26, 27]. These early innate immune responses are essential for viral recognition, restriction of viral replication, and priming of adaptive immunity [15, 28]. However, excessive or dysregulated TLR activation may also contribute to immunopathology and tissue damage [29, 30, 31, 32]. In JEV infection, uncontrolled production of proinflammatory cytokines and chemokines has been implicated in neuroinflammation and neuronal injury.

In addition to direct viral sensing, mosquito saliva can modulate host innate immune responses and enhance the pathogenicity of several flaviviruses. Activation of TLR2‐associated signaling pathways by mosquito salivary components has been shown to promote recruitment of virus‐permissive myeloid cells to the site of infection [33]. These findings highlight the importance of vector‐mediated modulation of innate immune pathways during the early stages of flaviviral infection.

A comprehensive understanding of host innate immune responses during JEV infection is essential for elucidating disease pathogenesis and advancing the development of effective therapeutic and preventive strategies. Although previous reviews have summarized the general immunopathogenesis and antiviral responses associated with JEV infection, recent studies have revealed increasingly complex interactions between TLR signaling, cytosolic PRR pathways, neuroinflammatory responses, autophagy, immune‐metabolic regulation, and viral immune evasion mechanisms [6, 11, 34, 35, 36]. In addition, accumulating evidence indicates that innate immune pathways may exert both protective antiviral and pathogenic neuroinflammatory effects in a context‐dependent manner.

This article is presented as a narrative review intended to summarize and critically discuss current knowledge regarding innate immune responses and viral immune evasion during JEV infection, with particular emphasis on TLR signaling and PRR crosstalk. Relevant literature was identified through searches of PubMed, Scopus, and Google Scholar using combinations of the keywords “Japanese encephalitis virus,” “innate immunity,” “Toll‐like receptor,” “TLR,” “RIG‐I,” “MDA5,” “interferon,” “autophagy,” “microRNA,” “immune evasion,” and “neuroinflammation.” Priority was given to peer‐reviewed original research articles and recent review articles published in English, particularly studies addressing mechanistic aspects of host–virus interactions, antiviral signaling pathways, immunopathogenesis, and translational implications relevant to JEV infection.

Accordingly, this review focuses on the integrated roles of TLR‐mediated and PRR‐associated innate immune responses during JEV infection, with emphasis on signaling crosstalk, cell type‐specific immune regulation within the CNS, viral immune evasion strategies targeting these pathways, and the translational potential of TLR‐targeted immunomodulators and vaccine adjuvants.

## 2 Innate Immune Response to JEV Infection

The innate immune system represents the first line of defense against JEV infection through recognition of viral components and infection‐associated danger signals by PRRs, including TLRs and retinoic acid‐inducible gene I (RIG‐I)‐like receptors (RLRs), leading to activation of antiviral and inflammatory signaling pathways [36, 37, 38]. Activation of these receptors initiates downstream signaling involving IRF3/7, NF‐κB, and type I IFNs, thereby promoting induction of antiviral effectors, cytokines, and chemokines that shape host defense and inflammatory responses during infection. Within the central nervous system (CNS), resident neural cells, including neurons, microglia, and astrocytes, together with infiltrating immune cells, contribute to antiviral defense and inflammatory responses during infection. Although these innate immune mechanisms are important for limiting viral replication, excessive or dysregulated activation may also contribute to neuroinflammation and CNS pathology.

### 2.1 PRR Sensing and Antiviral Signaling During JEV Infection

Neurons are capable of mounting intrinsic antiviral responses against JEV infection. Recognition of viral RNA by RIG‐I promotes interaction with STING and subsequent activation of type I IFN signaling pathways [37, 39]. Disruption of STING signaling reduces the expression of inflammatory mediators and enhances intracellular viral replication, whereas STING overexpression suppresses viral propagation, highlighting the importance of the RIG‐I–STING axis in neuronal antiviral defense [39]. Consistent with these findings, transcriptomic analyses of JEV‐infected neuronal cells demonstrated induction of IFN‐stimulated genes (ISGs), chemokine networks, and stress response pathways, supporting a role for neuron‐intrinsic RIG‐I and MDA5 signaling in antiviral immunity and neuropathogenesis [40].

Microglia, the resident immune cells of the CNS, represent major mediators of innate immune responses during JEV infection. Recognition of viral double‐stranded RNA through TLR3‐ and RIG‐I–dependent pathways promotes microglial activation and secretion of inflammatory cytokines and chemokines [41]. Increased expression of TLR2, TLR4, TLR9, IL‐6, IL‐17, MCP‐1, and RANTES has been reported in infected BV2 microglial and Neuro2A neuronal cells, together with reduced expression of IFN‐γ, IL‐21, and FoxP3, suggesting enhanced inflammatory activation accompanied by impaired immune regulation [42]. In addition, JEV‐induced RANTES production in glial cells occurs through extracellular signal‐regulated kinase (ERK)‐dependent NF‐κB and IL‐6 signaling pathways, thereby promoting immune cell recruitment and amplification of neuroinflammation [43]. Although these responses may contribute to viral clearance, sustained microglial activation and excessive inflammatory mediator production can also promote neuronal injury and CNS pathology [44].

Several TLRs appear to play distinct and context‐dependent roles during JEV infection. Genetic studies have shown that TLR3 polymorphisms, including the Leu412Phe variant, are associated with increased susceptibility to Japanese encephalitis, supporting the importance of early viral recognition in host defense [45]. Consistently, studies using TLR‐deficient mice demonstrated divergent effects of individual TLR pathways on viral control and neuroinflammation. TLR3 deficiency increased susceptibility to infection by enhancing viral replication, impairing type I IFN responses, and exacerbating CNS inflammation, whereas TLR4 deficiency conferred partial resistance associated with enhanced antiviral immune responses and reduced neuropathology [46]. TLR7 also contributes to antiviral defense by recognizing JEV‐derived single‐stranded RNA and promoting early IFN responses that restrict systemic viral spread [38]. Correspondingly, TLR7‐deficient mice exhibit elevated viral loads, while compensatory upregulation of TLR8 suggests partial functional redundancy between these receptors [47]. Pharmacological activation of TLR7 using imiquimod further improved survival in infected mice, supporting the protective role of this pathway [38].

Temporal regulation of TLR expression may additionally influence disease progression. Transcriptomic analyses of infected mouse brains revealed persistent TLR3 upregulation throughout infection, whereas expression of TLR1, TLR2, and TLR7 increased predominantly during later stages of disease [48]. These findings suggest that different TLR pathways may contribute variably to early antiviral defense and later inflammatory pathology. Furthermore, the JEV E protein has been shown to induce dose‐dependent TLR4 expression in HEK293T and A549 cells, leading to increased secretion of TNF‐α, IL‐1β, and IL‐6 and contributing to localized inflammatory response [49].

Additional PRRs and downstream signaling pathways also contribute to JEV‐induced innate immunity, mitochondrial damage during infection can release mitochondrial DNA, which activates TLR9 signaling and promotes expansion of myeloid‐derived suppressor cells [50]. Proteomic analyses further revealed activation of the TLR2–PI3K–AKT signaling pathway during early infection, suggesting that TLR2 signaling may contribute to both antiviral responses and inflammatory pathology [51]. Similarly, increased expression of TLR2 and MDA5 in neuronal cells and infected mouse brains indicates that PRR engagement may vary according to cell type and developmental stage [52].

Collectively, these findings indicate that PRR‐mediated responses during JEV infection are highly context‐dependent and may exert both antiviral and pathogenic effects. TLR3 and TLR7 signaling are generally associated with induction of type I IFNs and restriction of viral replication, particularly during the early stages of infection [38, 46]. In contrast, activation of TLR2‐ and TLR4‐associated pathways appears to contribute more prominently to inflammatory cytokine production, microglial activation, and amplification of neuroinflammatory responses [42, 46, 48, 51]. However, these effects are not strictly protective or pathogenic, as controlled inflammatory signaling may facilitate viral clearance whereas sustained activation can promote CNS injury. Functional outcomes also vary substantially according to cell type, viral strain, infection stage, and experimental model because neurons, microglia, macrophages, and peripheral immune cells differ in PRR expression profiles and downstream signaling capacity [45, 47, 52]. These observations underscore the complexity of PRR‐mediated immune regulation during JEV infection.

Bats are recognized as important reservoir hosts for numerous zoonotic viruses and possess unique antiviral immune adaptations that enable viral tolerance with limited inflammatory pathology, making bat‐derived epithelial cell models valuable for investigating conserved innate immune mechanisms relevant to flaviviral infection and JEV pathogenesis [53]. Accordingly, bat‐derived kidney epithelial cells have been used to examine host–virus interactions and antiviral signaling pathways that may contribute to JEV persistence and pathogenesis in mammalian hosts. In these cells, knockdown of TLR3, RIG‐I, or MDA5 reduced IFN‐β production and enhanced JEV replication, demonstrating the importance of these PRRs in restricting viral infection [54]. Given the context‐dependent roles of PRRs during JEV infection, Table 1 summarizes their reported ligands or activating stimuli, signaling adapters, downstream immune pathways, and proposed contributions to antiviral immunity and immunopathogenesis.

**TABLE 1:** Summary of pattern recognition receptors involved in JEV infection and their downstream signaling pathways.

PRR	Major ligand/sensed component during JEV infection	Signaling adapter(s)	Major downstream pathways/effects	Reported role in JEV infection	References
TLR2	Viral envelope‐associated components; inflammatory DAMPs	MyD88	NF‐κB activation, proinflammatory cytokine production	Contributes to inflammatory responses and neuroinflammation; activates PI3K–AKT signaling in microglia	[42, 51]
TLR3	Viral dsRNA replication intermediates	TRIF	IRF3, NF‐κB, type I IFN induction	Protective antiviral role; restricts viral replication and CNS dissemination	[18, 41, 45, 46]
TLR4	JEV envelope (E) protein	MyD88/TRIF	NF‐κB activation, IL‐6, TNF‐α, IL‐1β production	Promotes inflammatory responses; TLR4 deficiency associated with reduced neuroinflammation and improved survival	[42, 46, 49]
TLR7	Viral ssRNA	MyD88	IRF7 activation, type I IFN production	Promotes antiviral immunity and limits viral spread; imiquimod‐mediated activation improves survival	[19, 38]
TLR8	Viral ssRNA (putative/compensatory sensing)	MyD88	Inflammatory cytokine and IFN signaling	Compensatory upregulation observed in TLR7‐deficient mice	[47]
TLR9	Mitochondrial DNA/DAMPs released during infection	MyD88	NF‐κB activation, inflammatory signaling	Promotes expansion of myeloid‐derived suppressor cells during infection	[50]
RIG‐I	Cytosolic viral ssRNA with 5′‐triphosphate ends	MAVS/STING	IRF3/7 activation, type I IFN induction, ISG expression	Critical neuronal antiviral sensor limiting viral replication	[22, 37, 39]
MDA5	Viral dsRNA replication intermediates	MAVS	Type I IFN signaling, antiviral gene induction	Contributes to antiviral immunity in neurons and bat epithelial cells	[40, 54]

### 2.2 Cell Type‐Specific Innate Immune Responses and Immunopathology

Innate immune responses within the CNS involve coordinated interactions between resident neural cells and infiltrating peripheral immune cells. Transcriptomic profiling of JEV‐infected mouse brains demonstrated early activation of multiple antiviral pathways, including IFN signaling, ISGs, complement activation, protein‐processing pathways, and recruitment of immune cells such as natural killer cells, macrophages, and infiltrating leukocytes [48].

Neutrophils also contribute to the early immune response against JEV infection. Experimental depletion of neutrophils reduced CD8^+^ T‐cell infiltration into the brain and accelerated mortality in infected mice [55], indicating the presence of a functional neutrophil– CD8^+^ T‐cell axis important for antiviral defense within the CNS.

Recent studies using human brain organoids have provided additional insights into age‐dependent susceptibility to JEV infection. Following infection, robust induction of ISGs was observed; however, the magnitude and pattern of ISGs responses varied according to developmental stage [56]. These findings may partially explain the increased susceptibility and disease severity observed in children, in whom immature or dysregulated antiviral responses may contribute to enhanced neuropathogenesis.

Although many mechanistic insights into JEV‐induced innate immune responses and CNS immunopathology have been derived from mouse models and in vitro systems, several clinical studies support the relevance of these pathways in human disease. Elevated concentrations of proinflammatory cytokines and chemokines, including IL‐6, IL‐8, TNF‐α, IFN‐γ, MCP‐1, and CXCL10, have been detected in the serum and cerebrospinal fluid of patients with Japanese encephalitis and are frequently associated with disease severity and neurological complications [57, 58, 59, 60]. Increased expression of inflammatory mediators and markers of microglial activation in Japanese encephalitis patients further supports the contribution of dysregulated innate immune responses to neuropathogenesis [61]. In addition, genetic association studies have identified TLR3 polymorphisms as potential susceptibility factors for Japanese encephalitis in humans [45], highlighting the clinical relevance of PRR‐mediated antiviral signaling pathways. Nevertheless, despite these observations, direct characterization of PRR signaling dynamics, cell type‐specific immune responses, and innate immune regulation in human CNS tissues remains limited.

### 2.3 Host Modulatory Factors Regulating Innate Immune Responses

MicroRNAs (miRNAs), small non‐coding RNAs that regulate gene expression through post‐transcriptional gene silencing, also play important roles in modulating innate immune responses during JEV infection. Several miRNAs contribute to host antiviral defense and regulation of inflammatory signaling pathways. For example, downregulation of miR‐370 during infection enhances viral replication, whereas restoration of miR‐370 suppresses viral replication and modulates innate immune gene expression, suggesting an antiviral role for this miRNA [62]. Overexpression of miR‐466d‐3p inhibits IL‐1β expression and reduces viral replication [63]. Similarly, miR‐155 overexpression in human brain microglial cells suppresses JEV replication and modulates NF‐κB–regulated inflammatory signaling, indicating its role in antiviral immune regulation [64]. In addition, let‐7a/b miRNAs regulate microglial inflammatory responses through interactions with TLR7 and NOTCH signaling pathways, thereby enhancing NF‐κB–dependent TNF‐α production [65].

Multiple host factors modulate innate immune signaling and influence disease outcomes during JEV infection. Growth arrest–specific gene 6 (GAS6)–TAM receptors, particularly Axl, regulate antiviral and inflammatory responses in infected tissues [66]. GAS6–Axl signaling suppresses macrophage pyroptosis and enhances neuronal ISG expression through PI3K–Akt signaling, thereby limiting viral replication and tissue injury, whereas Axl‐deficient mice exhibit impaired antiviral responses and increased susceptibility to infection [67, 68].

Indoleamine 2,3‐dioxygenase (IDO), an immunoregulatory enzyme involved in tryptophan metabolism, suppresses type I IFN responses during JEV infection. Pharmacological inhibition of IDO restores PRR signaling and reduces viral dissemination [69]. Poly(ADP‐ribose) polymerase 1 (PARP1), a nuclear enzyme functioning in DNA repair and inflammatory signaling, also participates in JEV pathogenesis. Inhibition of PARP1 suppresses autophagy and reduces viral replication, suggesting a proviral role during infection [70]. Similarly, zinc‐finger antiviral protein restricts JEV replication, whereas depletion of this host factor enhances viral propagation [71]. Additionally, melatonin has been reported to attenuate neuroinflammation and suppress viral replication by interfering with the viral NS3 and NS5 proteins [72].

**FIGURE 1:**
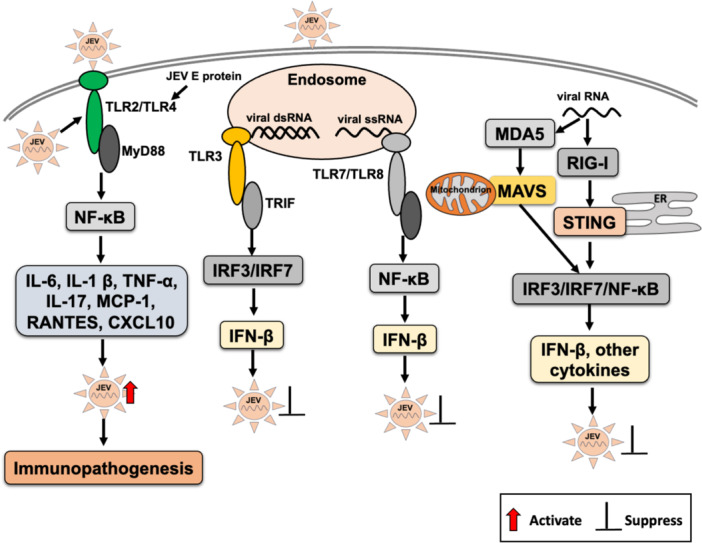
Innate immune response to Japanese encephalitis virus (JEV) infection. Red arrows indicate pathways that enhance JEV replication, while black blunted arrows indicate pathways that suppress or reduce viral replication. CXCL10, C‐X‐C motif chemokine ligand 10; ER, endoplasmic reticulum; IFN‐β, interferon beta; IL‐17, interleukin‐17; IL‐1β, interleukin‐1 beta; IL‐6, interleukin‐6; IRF, interferon regulatory factor; MAVS, mitochondrial antiviral signaling protein; MCP‐1, monocyte chemoattractant protein‐1; MDA5, melanoma differentiation‐associated protein 5; MyD88, myeloid differentiation primary response 88; NF‐κB, nuclear factor κ‐light‐chain‐enhancer of activated B cells; RANTES, regulated on activation, normal T cell expressed and secreted; RIG‐I, retinoic acid‐inducible gene I; STING, stimulator of interferon genes; TLR2/3/4/7/8, toll‐like receptors 2, 3, 4, 7, and 8; TNF‐α, tumor necrosis factor alpha; TRIF, TIR‐domain‐containing adapter‐inducing interferon‐β.

Collectively, these findings demonstrate that JEV infection induces a highly coordinated yet potentially pathogenic innate immune response involving PRR signaling, IFN induction, inflammatory cytokine production, and host regulatory mechanisms. While these responses are essential for restricting viral replication, excessive or dysregulated activation can contribute substantially to CNS inflammation and neurological damage. A schematic overview of the innate immune response to JEV infection is presented in Figure 1.

## 3 Inhibition of Innate Immune Response by JEV Infection

Japanese encephalitis virus employs multiple strategies to evade host innate immune defenses and create a cellular environment conducive to viral replication and spread. These evasion strategies primarily interfere with PRR‐mediated signaling, type I IFN production, inflammatory pathways, and antiviral effector functions. Importantly, many of these pathways are highly interconnected, allowing JEV to simultaneously suppress antiviral responses while dysregulating inflammatory signaling, thereby promoting viral persistence and contributing to neuropathogenesis.

### 3.1 Suppression of PRR Signaling and Type I IFN Responses

Type I IFN production is a central antiviral defense mechanism activated downstream of PRRs such as TLR3, RIG‐I, and MDA5. JEV interferes with multiple components of these signaling cascades to attenuate host antiviral immunity. The viral nonstructural protein NS1 functions as a key immune evasion factor by targeting DEAD‐box helicase 3 X‐linked (DDX3X), a cellular RNA helicase that facilitates IFN‐β transcription and promotes the expression of ISGs. The interaction of NS1 with DDX3X disrupts the latter's antiviral activity, suppressing IFN‐β signaling and enhancing viral replication [73]. JEV NS4B also antagonizes antiviral defense by inhibiting TLR3‐dependent pathways. TLR3 normally detects viral double‐stranded RNA within endosomes and activates the adapter molecule TRIF, leading to IRF3 and NF‐κB activation and subsequent IFN production. NS4B interferes with this pathway by targeting TRIF‐associated signaling events, thereby suppressing IFN‐β induction and dampening host antiviral responses [74]. Similarly, the viral NS5 protein inhibits the dsRNA‐triggered nuclear translocation of IRF3 and NF‐κB, two transcription factors essential for type I IFN induction and inflammatory cytokine production [75]. Collectively, these findings indicate that JEV suppresses innate antiviral defense through the coordinated inhibition of multiple PRR‐associated signaling cascades.

### 3.2 Modulation of Host miRNAs and Antiviral Signaling

MicroRNAs also play important roles in antiviral immunity, inflammatory signaling, and viral pathogenesis. JEV modulates host miRNA expression to suppress antiviral responses and facilitate viral replication. The JEV NS3 helicase impairs maturation of miRNA 466d‐3p by promoting arginine‐dependent degradation of precursor miRNAs, thereby enhancing IL‐1β–mediated neuroinflammation and viral replication [63]. JEV‐induced upregulation of miR‐301a further contributes to viral immune evasion by suppressing IFN production through targeting IRF1 and suppressor of cytokine signaling 5 (SOCS5), both of which are involved in antiviral signaling pathways [76].

Conflicting findings have been reported regarding the role of miR‐146a during JEV infection. In CHME3 human microglial cells, JEV‐induced miR‐146a expression suppressed TRAF6, IRAK1, IRAK2, and STAT1 signaling, decreased ISG expression, and enhanced viral replication [77]. In contrast, another in vitro study observed no significant effect of miR‐146a overexpression on JEV replication [64]. Although differences in multiplicity of infection may contribute to these discrepancies, cell type‐specific effects likely also play an important role. Because miRNA‐mediated regulation is highly dependent on cellular context and target gene expression profiles, the contradictory findings highlight the complexity of miRNA regulation during JEV infection and indicate that the precise role of miR‐146a in antiviral defense remains unresolved.

### 3.3 Autophagy‐Mediated Immune Modulation

Autophagy is a highly regulated cellular degradation pathway involved in cellular homeostasis, stress responses, and innate immunity. During viral infection, autophagy may exert either antiviral or proviral effects depending on the virus, infected cell type, and stage of infection. Increasing evidence suggests that JEV modulates autophagy to influence antiviral signaling; however, the overall consequences of autophagy regulation during JEV infection remain incompletely understood and are currently under active investigation [78, 79, 80].

Several studies indicate that JEV‐induced autophagy can suppress type I IFN responses and facilitate viral replication. Autophagy has been shown to limit activation of key antiviral signaling molecules, including MAVS and IRF3, thereby attenuating IFN signaling and promoting viral replication [81]. Consistently, autophagy‐deficient cells exhibit enhanced IFN activation and reduced viral replication, suggesting that JEV may exploit autophagic pathways to evade innate immune responses [81]. JEV‐mediated downregulation of SIRT2 may further contribute to autophagy‐associated immune modulation. SIRT2, a nicotinamide adenine dinucleotide (NAD + )‐dependent deacetylase involved in regulation of inflammatory signaling and cellular homeostasis, normally restrains NF‐κB activation [82, 83]. During JEV infection, reduced SIRT2 expression enhances NF‐κB‐mediated inflammatory responses and promotes Beclin‐1–associated autophagy, thereby creating a cellular environment favorable for viral replication [84]. Together, these findings suggest that inflammatory signaling and autophagy pathways may act cooperatively during infection.

However, the relationship between JEV and autophagy is likely more complex than a uniformly proviral interaction. Previous studies have also reported inhibitory effects of JEV on autophagy‐related pathways, indicating that the virus may differentially regulate autophagy depending on the stage of infection and cellular context [79, 85]. Consequently, the net virological and immunological outcome of autophagy modulation during JEV infection remains uncertain. This complexity has important therapeutic implications, as both autophagy induction and inhibition have been proposed as potential antiviral strategies, underscoring the need for further mechanistic studies before autophagy‐targeted interventions can be translated clinically.

### 3.4 Modulation of Host Antiviral Effector Molecules

JEV also manipulates host antiviral effector proteins to promote infection. Guanylate‐binding protein 1 (GBP1), an IFN‐inducible GTPase involved in antimicrobial and antiviral defense, was reported to be upregulated during JEV infection in HeLa cells [86]. Under IFN‐γ–primed conditions, GBP1 paradoxically suppressed STAT1‐mediated antiviral signaling, and enhanced viral replication [86]. Therefore, although the study suggests a potential proviral role for GBP1, additional validation in neural and in vivo models is required to determine its relevance to human JEV neuropathogenesis.

**FIGURE 2:**
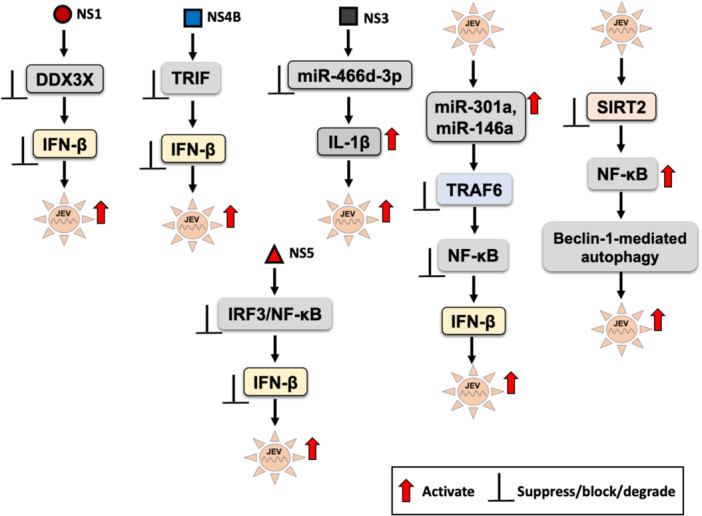
Mechanisms by which Japanese encephalitis virus (JEV) and its proteins modulate the host innate immune response to enhance viral replication. Red arrows indicate activation or induction of innate immune molecules or JEV replication, as appropriate. Black blunted arrows indicate suppression, inhibition, or degradation of host immune molecules, ultimately promoting viral replication.

Collectively, these findings demonstrate that JEV employs diverse and interconnected strategies to suppress innate immune signaling, impair type I IFN responses, manipulate host regulatory pathways, and create a cellular environment favorable for viral replication and persistence. A schematic overview of these immune evasion mechanisms is presented in Figure 2.

## 4 TLR Agonist–Mediated Enhancement of Antiviral and Vaccine‐Induced Immunity Against JEV

Because TLR signaling plays an important role in antiviral immunity and the induction of adaptive immune responses during JEV infection, TLR agonists have attracted considerable interest as immunomodulators and vaccine adjuvants. By activating innate immune pathways and enhancing antigen presentation, TLR agonists can improve both humoral and cellular immune responses, thereby potentially improving vaccine efficacy and durability [87, 88].

Several licensed JEV vaccines are currently available, including live‐attenuated, inactivated Vero cell‐derived, chimeric, and recombinant vaccine platforms [89]. Although these vaccines generally demonstrate favorable safety and protective efficacy profiles, limitations remain in certain settings, including waning immunity over time, the need for booster immunization, variable immunogenicity in young children, elderly individuals, and immunocompromised populations, as well as differences in long‐term neutralizing antibody responses among vaccine platforms [89, 90, 91]. These challenges have stimulated interest in improved adjuvant strategies capable of enhancing the magnitude, breadth, and persistence of vaccine‐induced immunity.

TLR agonists function as precision adjuvants by stimulating innate immune pathways that promote activation and maturation of antigen‐presenting cells, cytokine production, and induction of antigen‐specific B‐ and T‐cell responses [88, 90]. Although several studies have demonstrated that TLR agonists can enhance vaccine efficacy against flaviviruses such as West Nile virus [92], evidence specifically supporting their efficacy in licensed JEV vaccine platforms remains limited, and their application in JEV vaccine development requires further investigation.

Among the TLR agonists evaluated in the context of JEV, CpG oligodeoxynucleotides (CpG ODNs), which activate TLR9 signaling, and imiquimod, a TLR7/8 agonist, have demonstrated promising immunostimulatory properties in experimental vaccine studies. These agonists enhance innate immune activation and promote maturation of dendritic cells and other antigen‐presenting cells, thereby strengthening downstream adaptive immune responses [93]. Co‐administration of CpG ODNs with a JEV DNA vaccine significantly increased production of TNF‐α and Th1‐associated cytokines, including IFN‐γ and IL‐2, and generated antibody responses comparable to those induced by two doses of vaccine alone [94]. These findings suggest that CpG ODN may improve vaccine immunogenicity while potentially enabling antigen dose sparing.

Similarly, imiquimod‐mediated activation of TLR7 signaling has been associated with enhanced antiviral immune responses through induction of type I IFNs and promotion of Th1‐biased immunity. Because TLR7 signaling plays an important role in antiviral defense against JEV [38], incorporation of TLR7/8 agonists into future vaccine formulations may represent a promising strategy to enhance cellular immunity and long‐term protection [95]. In addition to CpG ODNs and imiquimod, other TLR agonist platforms targeting TLR3, TLR4, and combination adjuvant systems have shown immunostimulatory potential in experimental antiviral vaccines [96] and may also have relevance for next‐generation JEV vaccine development.

Overall, accumulating evidence suggests that TLR agonists could serve as valuable adjuncts for improving JEV vaccine immunogenicity and antiviral protection. However, translation of these approaches into clinical application requires careful consideration of several challenges, including optimization of agonist dose, route of administration, formulation stability, and long‐term safety. Because excessive TLR activation may amplify inflammatory responses and potentially exacerbate neuroinflammation or tissue injury, particularly in the CNS, balancing immunostimulatory efficacy with controlled immune activation remains critical. In addition, variability in host immune status, age‐dependent immune responses, and limited data from human clinical studies further complicate the development of TLR‐targeted interventions for JEV. Therefore, additional mechanistic studies and evaluation in clinically relevant animal models and human trials are needed to establish the safety, efficacy, and translational feasibility of TLR agonist‐based therapeutic and vaccine strategies against JEV.

## 5 Discussion

**FIGURE 3:**
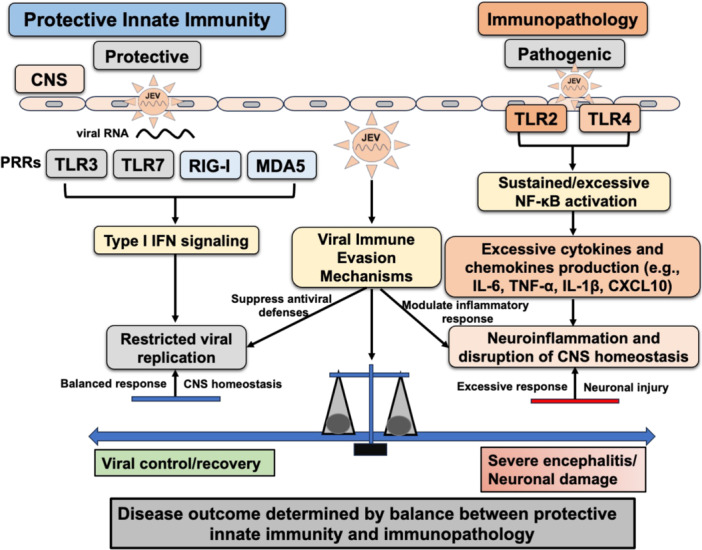
Balance between protective innate immunity and immunopathology during Japanese encephalitis virus (JEV) infection. JEV‐derived viral RNA is recognized by pattern recognition receptors (PRRs), including Toll‐like receptor 3 (TLR3), Toll‐like receptor 7 (TLR7), retinoic acid‐inducible gene I (RIG‐I), and melanoma differentiation‐associated protein 5 (MDA5), leading to activation of type I interferon (IFN) signaling and induction of interferon‐stimulated genes (ISGs). These antiviral responses restrict viral replication and contribute to central nervous system (CNS) homeostasis, promoting viral control and recovery. Conversely, activation of TLR2 and TLR4 stimulates sustained/excessive NF‐κB signaling, resulting in overproduction of pro‐inflammatory cytokines and chemokines, including IL‐6, TNF‐α, IL‐1β, and CXCL10. Excessive inflammatory responses drive neuroinflammation, disrupt CNS homeostasis, and contribute to neuronal injury and severe encephalitis. Simultaneously, JEV employs immune evasion mechanisms that suppress antiviral defenses and modulate inflammatory responses, thereby influencing both protective and pathogenic pathways. Overall, disease outcome is determined by the dynamic balance between protective innate immunity and immunopathology.

An emerging concept in JEV immunopathogenesis is that innate immune responses exert both protective and pathogenic effects, with disease outcome largely determined by the balance between antiviral defense and inflammation‐mediated tissue injury. Early recognition of viral components by PRRs, including endosomal TLR3 and TLR7, as well as cytosolic RIG‐I and MDA5, activates type I IFN signaling and ISG expression, thereby restricting viral replication and promoting protective adaptive immune responses [37, 38, 39, 41]. In contrast, JEV has evolved multiple immune evasion mechanisms that suppress antiviral signaling pathways while modulating host inflammatory responses to facilitate viral replication and persistence [73, 74, 75]. Moreover, activation of TLR2‐ and TLR4‐associated pathways under certain conditions may further amplify inflammatory pathology [42, 46, 49]. Sustained activation of microglia and infiltrating peripheral immune cells can subsequently drive excessive production of proinflammatory cytokines and chemokines, leading to disruption of CNS homeostasis, neuroinflammation, and neuronal injury [41, 44]. A schematic overview summarizing these interactions is presented in Figure 3.

Despite considerable advances in understanding PRR‐mediated signaling during JEV infection, several important mechanistic and translational questions remain unresolved. One major challenge is the context‐dependent nature of TLR signaling and its downstream effects, which vary according to cell type, viral strain, stage of infection, and experimental model [42, 48, 52]. Consequently, the molecular mechanisms that distinguish protective antiviral immunity from pathogenic inflammatory responses remain incompletely defined. Addressing these gaps will require future studies integrating temporal analyses, cell type‐specific experimental systems, and comparative evaluation of different JEV genotypes to better clarify how distinct PRR signaling pathways regulate CNS immunity during infection.

Another unresolved area involves the complex relationship between autophagy and innate immune signaling. Autophagy interacts closely with PRR pathways, including TLR signaling, and can modulate both IFN production and inflammatory responses [97, 98]. However, both proviral and antiviral effects of autophagy have been reported during JEV infection depending on the experimental context [81, 85, 99], indicating that its definitive role remains incompletely defined. Consequently, therapeutic modulation of autophagy may exert competing effects on viral replication, neuronal survival, and inflammatory signaling. Although autophagy‐targeting strategies have shown promise in experimental settings—such as enhancing JEV DNA vaccine immunogenicity through LC3‐associated antigen targeting [100], their translational feasibility and safety in humans require rigorous investigation.

Translating findings from experimental models to human clinical disease also remains a major bottleneck in the field. Much of the current mechanistic understanding is derived from knockout mouse models, immortalized cell lines, or non‐neuronal in vitro systems that fail to fully recapitulate the complexity of human CNS infection, blood–brain barrier dynamics, or age‐dependent immune responses [34, 48, 56]. Additionally, heterogeneity among patients with respect to viral exposure history, vaccination status, host genetics, and inflammatory states complicates the identification of reliable immune correlates of protection or disease severity [45, 89, 90]. Although elevated levels of inflammatory cytokines and chemokines have been documented in patients with Japanese encephalitis, direct characterization of PRR signaling dynamics and cell‐type‐specific immune responses within human CNS tissues remains sparse [38, 101].

Future research should therefore prioritize clinically relevant and translational approaches, including studies using human clinical specimens, advanced brain organoid systems, single‐cell and spatial transcriptomics, and integrated multi‐omics analyses [40, 56]. Greater emphasis is also needed to identify predictive immune biomarkers associated with disease severity, neurological sequelae, and therapeutic outcomes in patients [45]. In parallel, further evaluation of TLR‐targeted immunomodulators and host‐directed therapeutic strategies will be essential to determine their safety, efficacy, and clinical applicability [88]. Addressing these unresolved questions will clarify the precise mechanisms governing protective vs. pathogenic innate immune responses, ultimately facilitating the development of more effective antiviral and immunomodulatory interventions against JEV infection.

## 6 Conclusion

JEV remains a significant global health threat because of its neuroinvasive potential and its capacity to induce severe neurological sequelae. Innate immune responses play a critical role in host defense against JEV infection and in shaping disease progression within the CNS. Recognition of viral components by PRRs, particularly TLRs and cytosolic RNA sensors, initiates signaling cascades that drive the production of type I IFNs, proinflammatory cytokines, and ISGs. While these early responses are essential for limiting viral replication and coordinating adaptive immunity, the same inflammatory mechanisms can promote profound neuroinflammation and neuronal injury when excessively or chronically activated.

To counteract host defenses, JEV has evolved a diverse array of strategies to evade or manipulate innate immune signaling, including the inhibition of IFN pathways, modulation of host miRNAs, and exploitation of cellular processes like autophagy. These countermeasures underscore the dynamic interplay between host antiviral immunity and viral survival tactics that ultimately dictate clinical severity.

Consequently, advances in decoding the molecular mechanisms governing PRR signaling and viral evasion have profound implications for therapeutic and vaccine development. In particular, TLR agonists represent promising immunomodulatory tools capable of enhancing antiviral pathways and improving vaccine‐induced immunity. Future research aimed at clarifying the precise thresholds separating protective immunity from pathogenic neuroinflammation will be critical for developing targeted strategies to prevent and control JEV infection.

## Author Contributions

**Mohammad Enamul Hoque Kayesh:** conceptualization, writing, and editing. **Michinori Kohara:** conceptualization, writing, and editing. **Kyoko Tsukiyama‐Kohara:** conceptualization, writing, and editing. All authors read and approved the final manuscript.

## Conflicts of Interest

The authors declare no conflicts of interest.

## Data Availability

The data that support the findings of this study are available from the corresponding author upon reasonable request.

## References

[1] G. Campbell , S. Hills , M. Fischer , et al., “Estimated Global Incidence of Japanese Encephalitis: A Systematic Review,” Bulletin of the World Health Organization 89, no. 10 (2011): 766–774, 774A–774E. 22084515 10.2471/BLT.10.085233 PMC3209971

[2] J. S. Mackenzie , D. J. Gubler , and L. R. Petersen , “Emerging Flaviviruses: The Spread and Resurgence of Japanese Encephalitis, West Nile and Dengue Viruses,” Nature Medicine 10, no. 12 Suppl (2004): S98–S109. 10.1038/nm1144 15577938

[3] “Japanese Encephalitis,” World Health Organization (WHO), accessed May 14, 2026, https://www.who.int/news-room/fact-sheets/detail/japanese-encephalitis.

[4] “Japanese Encephalitis Virus,” Centers for Disease Control and Prevention (CDC), accessed May 14, 2026, https://www.cdc.gov/japaneseencephalitis/about/index.html.

[5] S. I. Yun and Y. M. Lee , “Japanese Encephalitis: The Virus and Vaccines,” Human Vaccines & Immunotherapeutics 10, no. 2 (2014): 263–279. 24161909 10.4161/hv.26902 PMC4185882

[6] S. Kumar , A. Verma , P. Yadav , et al., “Molecular Pathogenesis of Japanese Encephalitis and Possible Therapeutic Strategies,” Archives of Virology 167, no. 9 (2022): 1739–1762. 35654913 10.1007/s00705-022-05481-z PMC9162114

[7] A. J. Schuh , M. J. Ward , A. J. Leigh Brown , and A. D. T. Barrett , “Phylogeography of Japanese Encephalitis Virus: Genotype Is Associated With Climate,” PLoS Neglected Tropical Diseases 7, no. 8 (2013): e2411. 24009790 10.1371/journal.pntd.0002411 PMC3757071

[8] K. L. Mansfield , L. M. Hernández‐Triana , A. C. Banyard , A. R. Fooks , and N. Johnson , “Japanese Encephalitis Virus Infection, Diagnosis and Control in Domestic Animals,” Veterinary Microbiology 201 (2017): 85–92. 28284628 10.1016/j.vetmic.2017.01.014

[9] S. Edache , A. L. Dixon , A. R. S. Oliveira , et al., “Mosquito Vector Competence for Japanese Encephalitis Virus: A Systematic Review and Meta‐Analysis Update,” Parasites & Vectors 18, no. 1 (2025): 191. 40420304 10.1186/s13071-025-06843-7 PMC12107883

[10] K. J. N. Kishore , M. R. Praharaj , G. N. Tanuj , et al., “Tracing the Evolutionary Trajectory of Japanese Encephalitis Virus Across Hosts and Countries,” Scientific Reports 15, no. 1 (2025): 35061. 41062522 10.1038/s41598-025-03277-0 PMC12508474

[11] S. Kumar , R. Nyodu , K. Mv , and K. Ss , “Pathogenesis and Host Immune Response During Japanese Encephalitis Virus Infection.” in *Innate Immunity in Health and Disease* (IntechOpen, 2021), 10.5772/intechopen.98947.

[12] M. S. Diamond and T. D. Kanneganti , “Innate Immunity: The First Line of Defense Against SARS‐CoV‐2,” Nature Immunology 23, no. 2 (2022): 165–176. 35105981 10.1038/s41590-021-01091-0 PMC8935980

[13] T. Kawai , M. Ikegawa , D. Ori , and S. Akira , “Decoding Toll‐Like Receptors: Recent Insights and Perspectives in Innate Immunity,” Immunity 57, no. 4 (2024): 649–673. 38599164 10.1016/j.immuni.2024.03.004

[14] S. M. Lim , P. Koraka , A. D. M. E. Osterhaus , and B. E. E. Martina , “West Nile Virus: Immunity and Pathogenesis,” Viruses 3, no. 6 (2011): 811–828. 21994755 10.3390/v3060811 PMC3185772

[15] S. N. Lester and K. Li , “Toll‐Like Receptors in Antiviral Innate Immunity,” Journal of Molecular Biology 426, no. 6 (2014): 1246–1264. 24316048 10.1016/j.jmb.2013.11.024 PMC3943763

[16] T. Kawasaki and T. Kawai , “Toll‐Like Receptor Signaling Pathways,” Frontiers in Immunology 5 (2014): 461. 25309543 10.3389/fimmu.2014.00461 PMC4174766

[17] A. Chaturvedi and S. K. Pierce , “How Location Governs Toll‐Like Receptor Signaling,” Traffic 10, no. 6 (2009): 621–628. 19302269 10.1111/j.1600-0854.2009.00899.x PMC2741634

[18] L. Alexopoulou , A. C. Holt , R. Medzhitov , and R. A. Flavell , “Recognition of Double‐Stranded RNA and Activation of NF‐κB by Toll‐Like Receptor 3,” Nature 413, no. 6857 (2001): 732–738. 11607032 10.1038/35099560

[19] S. S. Diebold , T. Kaisho , H. Hemmi , S. Akira , and C. Reis e Sousa , “Innate Antiviral Responses by Means of TLR7‐Mediated Recognition of Single‐Stranded RNA,” Science 303, no. 5663 (2004): 1529–1531. 14976261 10.1126/science.1093616

[20] M. H. Heim and R. Thimme , “Innate and Adaptive Immune Responses in HCV Infections,” Journal of Hepatology 61, no. 1 Suppl (2014): S14–S25. 25443342 10.1016/j.jhep.2014.06.035

[21] S. M. Y. Lee , K. H. Kok , M. Jaume , et al., “Toll‐Like Receptor 10 Is Involved in Induction of Innate Immune Responses to Influenza Virus Infection,” Proceedings of the National Academy of Sciences 111, no. 10 (2014): 3793–3798. 10.1073/pnas.1324266111 PMC3956146 24567377

[22] T. Kawai and S. Akira , “Toll‐Like Receptor and RIG‐1‐Like Receptor Signaling,” Annals of the New York Academy of Sciences 1143 (2008): 1–20. 19076341 10.1196/annals.1443.020

[23] M. S. Lee and Y. J. Kim , “Signaling Pathways Downstream of Pattern‐Recognition Receptors and Their Cross Talk,” Annual Review of Biochemistry 76 (2007): 447–480. 10.1146/annurev.biochem.76.060605.122847 17328678

[24] K. A. Fitzgerald and J. C. Kagan , “Toll‐Like Receptors and the Control of Immunity,” Cell 180, no. 6 (2020): 1044–1066. 32164908 10.1016/j.cell.2020.02.041 PMC9358771

[25] S. Akira , K. Takeda , and T. Kaisho , “Toll‐Like Receptors: Critical Proteins Linking Innate and Acquired Immunity,” Nature Immunology 2, no. 8 (2001): 675–680. 11477402 10.1038/90609

[26] R. Medzhitov and C. Janeway, Jr. , “Innate Immunity,” New England Journal of Medicine 343, no. 5 (2000): 338–344. 10922424 10.1056/NEJM200008033430506

[27] T. H. Mogensen , “Pathogen Recognition and Inflammatory Signaling in Innate Immune Defenses,” Clinical Microbiology Reviews 22, no. 2 (2009): 240–273, Table of Contents. 19366914 10.1128/CMR.00046-08 PMC2668232

[28] M. Carty , C. Guy , and A. G. Bowie , “Detection of Viral Infections by Innate Immunity,” Biochemical Pharmacology 183 (2021): 114316. 33152343 10.1016/j.bcp.2020.114316

[29] M. E. H. Kayesh , M. Kohara , and K. Tsukiyama‐Kohara , “An Overview of Recent Insights Into the Response of TLR to SARS‐CoV‐2 Infection and the Potential of TLR Agonists as SARS‐CoV‐2 Vaccine Adjuvants,” Viruses 13, no. 11 (2021): 2302. 34835108 10.3390/v13112302 PMC8622245

[30] N. Modhiran , D. Watterson , D. A. Muller , et al., “Dengue Virus NS1 Protein Activates Cells Via Toll‐Like Receptor 4 and Disrupts Endothelial Cell Monolayer Integrity,” Science Translational Medicine 7, no. 304 (2015): 304ra142. 10.1126/scitranslmed.aaa3863 26355031

[31] B. Huang , J. Zhao , J. C. Unkeless , Z. H. Feng , and H. Xiong , “TLR Signaling by Tumor and Immune Cells: A Double‐Edged Sword,” Oncogene 27, no. 2 (2008): 218–224. 18176603 10.1038/sj.onc.1210904

[32] S. Yokota , T. Okabayashi , and N. Fujii , “The Battle Between Virus and Host: Modulation of Toll‐Like Receptor Signaling Pathways by Virus Infection,” Mediators of Inflammation 2010 (2010): 184328. 20672047 10.1155/2010/184328 PMC2903949

[33] T. Suzuki , Y. Miyata , S. Haga , et al., “Selective TLR Ligand Stimulation Enhances In Vivo Mosquito‐Borne Flavivirus Pathogenicity,” Cell Reports 44, no. 9 (2025): 116210. 40886313 10.1016/j.celrep.2025.116210

[34] S. K. Unni , D. Růžek , C. Chhatbar , R. Mishra , M. K. Johri , and S. K. Singh , “Japanese Encephalitis Virus: From Genome to Infectome,” Microbes and Infection 13, no. 4 (2011): 312–321. 21238600 10.1016/j.micinf.2011.01.002

[35] V. Deretic , T. Saitoh , and S. Akira , “Autophagy in Infection, Inflammation and Immunity,” Nature Reviews Immunology 13, no. 10 (2013): 722–737. 10.1038/nri3532 PMC5340150 24064518

[36] O. Takeuchi and S. Akira , “Pattern Recognition Receptors and Inflammation,” Cell 140, no. 6 (2010): 805–820. 20303872 10.1016/j.cell.2010.01.022

[37] A. Nazmi , K. Dutta , and A. Basu , “RIG‐I Mediates Innate Immune Response in Mouse Neurons Following Japanese Encephalitis Virus Infection,” PLoS One 6, no. 6 (2011): e21761. 21738791 10.1371/journal.pone.0021761 PMC3128083

[38] A. Nazmi , S. Mukherjee , K. Kundu , et al., “TLR7 Is a Key Regulator of Innate Immunity Against Japanese Encephalitis Virus Infection,” Neurobiology of Disease 69 (2014): 235–247. 24909816 10.1016/j.nbd.2014.05.036

[39] A. Nazmi , R. Mukhopadhyay , K. Dutta , and A. Basu , “STING Mediates Neuronal Innate Immune Response Following Japanese Encephalitis Virus Infection,” Scientific Reports 2 (2012): 347. 22470840 10.1038/srep00347 PMC3317237

[40] S. P. Yu , K. C. Ong , D. Perera , and K. T. Wong , “Neuronal Transcriptomic Responses to Japanese Encephalitis Virus Infection With a Special Focus on Chemokine CXCL11 and Pattern Recognition Receptors RIG‐1 and MDA5,” Virology 527 (2019): 107–115. 30481615 10.1016/j.virol.2018.10.015

[41] R. Jiang , J. Ye , B. Zhu , Y. Song , H. Chen , and S. Cao , “Roles of TLR3 and RIG‐I in Mediating the Inflammatory Response in Mouse Microglia Following Japanese Encephalitis Virus Infection,” Journal of Immunology Research 2014 (2014): 787023. 25101306 10.1155/2014/787023 PMC4101954

[42] M. Shukla , A. Garg , T. N. Dhole , and R. Chaturvedi , “Exaggerated Levels of Some Specific TLRs, Cytokines and Chemokines in Japanese Encephalitis Infected BV2 and Neuro 2A Cell Lines Associated With Worst Outcome,” Virology Journal 20, no. 1 (2023): 16. 36707891 10.1186/s12985-023-01966-8 PMC9881527

[43] C. J. Chen , J. H. Chen , S. Y. Chen , S. L. Liao , and S. L. Raung , “Upregulation of RANTES Gene Expression in Neuroglia by Japanese Encephalitis Virus Infection,” Journal of Virology 78, no. 22 (2004): 12107–12119. 15507597 10.1128/JVI.78.22.12107-12119.2004 PMC525064

[44] A. Ghoshal , S. Das , S. Ghosh , et al., “Proinflammatory Mediators Released by Activated Microglia Induces Neuronal Death in Japanese Encephalitis,” GLIA 55, no. 5 (2007): 483–496. 17203475 10.1002/glia.20474

[45] S. Biyani , R. K. Garg , A. Jain , et al., “Toll‐Like Receptor‐3 Gene Polymorphism in Patients With Japanese Encephalitis,” Journal of Neuroimmunology 286 (2015): 71–76. 26298326 10.1016/j.jneuroim.2015.07.010

[46] Y. W. Han , J. Y. Choi , E. Uyangaa , et al., “Distinct Dictation of Japanese Encephalitis Virus‐Induced Neuroinflammation and Lethality Via Triggering TLR3 and TLR4 Signal Pathways,” PLoS Pathogens 10, no. 9 (2014): e1004319. 25188232 10.1371/journal.ppat.1004319 PMC4154777

[47] M. Awais , K. Wang , X. Lin , et al., “TLR7 Deficiency Leads to TLR8 Compensative Regulation of Immune Response Against JEV in Mice,” Frontiers in Immunology 8 (2017): 160. 28265274 10.3389/fimmu.2017.00160 PMC5316529

[48] N. Gupta and P. L. Rao , “Transcriptomic Profile of Host Response in Japanese Encephalitis Virus Infection,” Virology Journal 8 (2011): 92. 21371334 10.1186/1743-422X-8-92 PMC3058095

[49] Y. Gao , Q. Sheng , X. Shi , et al., “Japanese Encephalitis Virus Envelope Protein Activates the TLR4/NF‑κB Pathway to Induce Testicular Inflammation,” Veterinary Microbiology 316 (2026): 110969. 41806590 10.1016/j.vetmic.2026.110969

[50] T. Lian , W. Zhang , H. Su , et al., “TLR9 Promotes Monocytic Myeloid‐Derived Suppressor Cell Induction During JEV Infection,” Microbes and Infection 26, no. 5–6 (2024): 105336. 38724001 10.1016/j.micinf.2024.105336

[51] G. Zhao , Y. Gao , J. Zhang , et al., “Toll‐Like Receptor 2 Signaling Pathway Activation Contributes to a Highly Efficient Inflammatory Response in Japanese Encephalitis Virus‐Infected Mouse Microglial Cells by Proteomics,” Frontiers in Microbiology 13 (2022): 989183. 36171749 10.3389/fmicb.2022.989183 PMC9511957

[52] P. R. Fadnis , V. Ravi , A. Desai , L. Turtle , and T. Solomon , “Innate Immune Mechanisms in Japanese Encephalitis Virus Infection: Effect on Transcription of Pattern Recognition Receptors in Mouse Neuronal Cells and Brain Tissue,” Viral Immunology 26, no. 6 (2013): 366–377. 24236856 10.1089/vim.2013.0016 PMC4649254

[53] A. T. Irving , M. Ahn , G. Goh , D. E. Anderson , and L. F. Wang , “Lessons From the Host Defences of Bats, a Unique Viral Reservoir,” Nature 589, no. 7842 (2021): 363–370. 33473223 10.1038/s41586-020-03128-0

[54] R. Tarigan , H. Shimoda , K. C. C. Doysabas , M. Ken , A. Iida , and E. Hondo , “Role of Pattern Recognition Receptors and Interferon‐Beta in Protecting Bat Cell Lines From Encephalomyocarditis Virus and Japanese Encephalitis Virus Infection,” Biochemical and Biophysical Research Communications 527, no. 1 (2020): 1–7. 32446351 10.1016/j.bbrc.2020.04.060 PMC7177169

[55] R. Soni , P. Jena , B. Kusuma , and A. Banerjee , “Neutrophil Depletion at the Early Stage of Japanese Encephalitis Virus Infection Affects CD8+ T Cell Infiltration into the Mouse Brain and Causes Severe Encephalitis,” Frontiers in Immunology 16 (2026): 1748085. 41646960 10.3389/fimmu.2025.1748085 PMC12867888

[56] B. Zhang , Y. He , Y. Xu , et al., “Differential Antiviral Immunity to Japanese Encephalitis Virus in Developing Cortical Organoids,” Cell Death & Disease 9, no. 7 (2018): 719. 29915260 10.1038/s41419-018-0763-y PMC6006338

[57] A. Mathur , R. Kulshreshtha , and A. Singh , “Secretion of the Chemokine Interleukin‐8 During Japanese Encephalitis Virus Infection,” Journal of Medical Microbiology 49, no. 7 (2000): 607–612. 10882085 10.1099/0022-1317-49-7-607

[58] T. Solomon , “Neurological Aspects of Tropical Disease: Japanese Encephalitis,” Journal of Neurology, Neurosurgery & Psychiatry 68, no. 4 (2000): 405–415. 10727474 10.1136/jnnp.68.4.405 PMC1736874

[59] P. M. Winter , N. M. Dung , H. T. Loan , et al., “Proinflammatory Cytokines and Chemokines in Humans With Japanese Encephalitis,” Journal of Infectious Diseases 190, no. 9 (2004): 1618–1626. 15478067 10.1086/423328

[60] R. Kumar , A. Mathur , A. Kumar , S. Sharma , S. Chakraborty , and U. C. Chaturvedi , “Clinical Features & Prognostic Indicators of Japanese Encephalitis in Children in Lucknow (India),” Indian Journal of Medical Research 91 (1990): 321–327. 2176644

[61] M. K. Mishra and A. Basu , “Minocycline Neuroprotects, Reduces Microglial Activation, Inhibits Caspase 3 Induction, and Viral Replication Following Japanese Encephalitis,” Journal of Neurochemistry 105, no. 5 (2008): 1582–1595. 18208541 10.1111/j.1471-4159.2008.05238.x

[62] W. Li , P. Cheng , S. Nie , and W. Cui , “miR‐370 Mimic Inhibits Replication of Japanese Encephalitis Virus in Glioblastoma Cells,” Neuropsychiatric Disease and Treatment 12 (2016): 2411–2417. 27703358 10.2147/NDT.S113236 PMC5036624

[63] H. Jiang , L. Bai , L. Ji , et al., “Degradation of MicroRNA miR‐466d‐3p by Japanese Encephalitis Virus NS3 Facilitates Viral Replication and Interleukin‐1β Expression,” Journal of Virology 94, no. 15 (2020): e00294‐20. 32461319 10.1128/JVI.00294-20 PMC7375366

[64] S. Pareek , S. Roy , B. Kumari , P. Jain , A. Banerjee , and S. Vrati , “MiR‐155 Induction in Microglial Cells Suppresses Japanese Encephalitis Virus Replication and Negatively Modulates Innate Immune Responses,” Journal of Neuroinflammation 11 (2014): 97. 24885259 10.1186/1742-2094-11-97 PMC4050406

[65] S. Mukherjee , I. Akbar , B. Kumari , S. Vrati , A. Basu , and A. Banerjee , “Japanese Encephalitis Virus‐Induced let‐7a/b Interacted With the NOTCH‐TLR7 Pathway in Microglia and Facilitated Neuronal Death Via Caspase Activation,” Journal of Neurochemistry 149, no. 4 (2019): 518–534. 30556910 10.1111/jnc.14645

[66] B. Hurtado and P. de Frutos , “GAS6 in Systemic Inflammatory Diseases: With and Without Infection,” Critical Care 14, no. 5 (2010): 1003. 21067537 10.1186/cc9263 PMC3219265

[67] Z. Y. Wang , Z. D. Zhen , D. Y. Fan , et al., “Axl Deficiency Promotes the Neuroinvasion of Japanese Encephalitis Virus by Enhancing IL‐1α Production From Pyroptotic Macrophages,” Journal of Virology 94, no. 17 (2020): e00602‐20. 32611752 10.1128/JVI.00602-20 PMC7431807

[68] J. Yang , M. Li , M. Yuan , et al., “Axl(‐/‐) Neurons Promote JEV Infection by Dampening the Innate Immunity,” Virus Research 307 (2022): 198605. 34662681 10.1016/j.virusres.2021.198605

[69] S. B. Kim , J. Y. Choi , E. Uyangaa , et al., “Blockage of Indoleamine 2,3‐Dioxygenase Regulates Japanese Encephalitis Via Enhancement of Type I/II IFN Innate and Adaptive T‐Cell Responses,” Journal of Neuroinflammation 13, no. 1 (2016): 79. 27090635 10.1186/s12974-016-0551-5 PMC4835894

[70] P. A. Desingu , S. Mishra , L. Dindi , et al., “PARP1 Inhibition Protects Mice Against Japanese Encephalitis Virus Infection,” Cell Reports 42, no. 9 (2023): 113103. 37676769 10.1016/j.celrep.2023.113103

[71] H. P. Chiu , H. Chiu , C. F. Yang , et al., “Inhibition of Japanese Encephalitis Virus Infection by the Host Zinc‐Finger Antiviral Protein,” PLoS Pathogens 14, no. 7 (2018): e1007166. 30016363 10.1371/journal.ppat.1007166 PMC6049953

[72] K. Kitidee , A. Samutpong , N. Pakpian , et al., “Antiviral Effect of Melatonin on Japanese Encephalitis Virus Infection Involves Inhibition of Neuronal Apoptosis and Neuroinflammation in SH‐SY5Y Cells,” Scientific Reports 13, no. 1 (2023): 6063. 37055489 10.1038/s41598-023-33254-4 PMC10099015

[73] H. Dong , Y. Hao , J. Wang , D. Chen , S. Xu , and W. Ruan , “Japanese Encephalitis Virus NS1 Inhibits IFN‐β Production by Interacting With DDX3X,” Journal of Virology 99, no. 5 (2025): e0007725. 40231822 10.1128/jvi.00077-25 PMC12090713

[74] Q. Zeng , J. Liu , Z. Li , et al., “Japanese Encephalitis Virus NS4B Inhibits Interferon Beta Production by Targeting TLR3 and TRIF,” Veterinary Microbiology 284 (2023): 109849. 37597377 10.1016/j.vetmic.2023.109849

[75] J. Ye , Z. Chen , Y. Li , et al., “Japanese Encephalitis Virus NS5 Inhibits Type I Interferon (IFN) Production by Blocking the Nuclear Translocation of IFN Regulatory Factor 3 and NF‐κB,” Journal of Virology 91, no. 8 (2017): e00039‐17. 28179530 10.1128/JVI.00039-17 PMC5375679

[76] B. Hazra , K. L. Kumawat , and A. Basu , “The Host MicroRNA miR‐301a Blocks the IRF1‐Mediated Neuronal Innate Immune Response to Japanese Encephalitis Virus Infection,” Science Signaling 10, no. 466 (2017): eaaf5185. 28196914 10.1126/scisignal.aaf5185

[77] N. Sharma , R. Verma , K. L. Kumawat , A. Basu , and S. K. Singh , “miR‐146a Suppresses Cellular Immune Response During Japanese Encephalitis Virus JaOArS982 Strain Infection in Human Microglial Cells,” Journal of Neuroinflammation 12 (2015): 30. 25889446 10.1186/s12974-015-0249-0 PMC4355369

[78] D. Sir and J. James Ou , “Autophagy in Viral Replication and Pathogenesis,” Molecules and Cells 29, no. 1 (2010): 1–8. 20077024 10.1007/s10059-010-0014-2 PMC3115743

[79] P. Y. Ke , “The Multifaceted Roles of Autophagy in Flavivirus‐Host Interactions,” International Journal of Molecular Sciences 19, no. 12 (2018): 3940. 30544615 10.3390/ijms19123940 PMC6321027

[80] J. K. Li , J. J. Liang , C. L. Liao , and Y. L. Lin , “Autophagy Is Involved in the Early Step of Japanese Encephalitis Virus Infection,” Microbes and Infection 14, no. 2 (2012): 159–168. 21946213 10.1016/j.micinf.2011.09.001

[81] R. Jin , W. Zhu , S. Cao , et al., “Japanese Encephalitis Virus Activates Autophagy as a Viral Immune Evasion Strategy,” PLoS One 8, no. 1 (2013): e52909. 23320079 10.1371/journal.pone.0052909 PMC3540057

[82] K. M. Rothgiesser , S. Erener , S. Waibel , B. Lüscher , and M. O. Hottiger , “SIRT2 Regulates NF‐κB‐Dependent Gene Expression Through Deacetylation of p65 Lys310,” Journal of Cell Science 123, no. Pt 24 (2010): 4251–4258. 21081649 10.1242/jcs.073783

[83] S. Roychowdhury , A. Gandhirajan , C. Kibler , X. Wang , and V. Vachharajani , “Sirtuin 2 Dysregulates Autophagy in High‐Fat‐Exposed Immune‐Tolerant Macrophages,” Cells 10, no. 4 (2021): 731. 33810233 10.3390/cells10040731 PMC8066127

[84] P. A. Desingu , L. Dindi , K. Murugasamy , et al., “SIRT2 Protects Against Japanese Encephalitis Virus Infection in Mice,” eLife 14 (2025): RP106778.

[85] M. Sharma , S. Bhattacharyya , M. Nain , et al., “Japanese Encephalitis Virus Replication Is Negatively Regulated by Autophagy and Occurs on LC3‐I‐ and EDEM1‐Containing Membranes,” Autophagy 10, no. 9 (2014): 1637–1651. 25046112 10.4161/auto.29455 PMC4206540

[86] S. Chhabra , K. B. Sharma , and M. Kalia , “Human Guanylate‐Binding Protein 1 Positively Regulates Japanese Encephalitis Virus Replication in an Interferon Gamma Primed Environment,” Frontiers in Cellular and Infection Microbiology 12 (2022): 832057. 35663470 10.3389/fcimb.2022.832057 PMC9160567

[87] F. Shao , X. Zhu , M. Yi , et al., “TLR Agonists as Adjuvants for Viral Vaccines: Mechanisms, Applications, and Future Directions,” Frontiers in Microbiology 16 (2026): 1740572. 41568029 10.3389/fmicb.2025.1740572 PMC12815852

[88] M. E. H. Kayesh , M. Kohara , and K. Tsukiyama‐Kohara , “TLR Agonists as Vaccine Adjuvants in the Prevention of Viral Infections: An Overview,” Frontiers in Microbiology 14 (2023): 1249718. 38179453 10.3389/fmicb.2023.1249718 PMC10764465

[89] D. Wu , Q. Yin , Y. Zhang , et al., “Immunogenicity, Immune Persistence, and Safety of Japanese Encephalitis Vaccine Schedules Among Adults in Ningxia, China,” NPJ Vaccines 10, no. 1 (2025): 222. 41168239 10.1038/s41541-025-01270-2 PMC12575666

[90] J. Y. Park and H. M. Lee , “Advances and Challenges in Vaccination and Therapeutic Strategies Against Japanese Encephalitis Virus,” Pathogens 14, no. 12 (2025): 1204. 41471160 10.3390/pathogens14121204 PMC12736247

[91] Y. L. Hu and P. I. Lee , “Safety of Japanese Encephalitis Vaccines,” Human Vaccines & Immunotherapeutics 17, no. 11 (2021): 4259–4264. 34613870 10.1080/21645515.2021.1969852 PMC8828133

[92] N. Van Hoeven , S. Wiley , E. Gage , et al., “A Combination of TLR‐4 Agonist and Saponin Adjuvants Increases Antibody Diversity and Protective Efficacy of a Recombinant West Nile Virus Antigen,” NPJ Vaccines 3 (2018): 39. 30302281 10.1038/s41541-018-0077-1 PMC6158298

[93] X. Zang , G. Li , J. Zhu , X. Dong , and Y. Zhai , “Evaluation of the Adjuvant Effect of Imiquimod and CpG ODN 1826 in Chimeric DNA Vaccine Against Japanese Encephalitis,” International Immunopharmacology 140 (2024): 112816. 39083930 10.1016/j.intimp.2024.112816

[94] J. N. Sidhapuriwala , S. P. Sivalingam , J. Lu , and S. M. Moochhala , “Immunomodulation of Japanese Encephalitis Vaccine Through CpG Oligodeoxynucleotides in Mice,” Scandinavian Journal of Immunology 64, no. 4 (2006): 370–375. 16970676 10.1111/j.1365-3083.2006.01793.x

[95] H. Hemmi , T. Kaisho , O. Takeuchi , et al., “Small Anti‐Viral Compounds Activate Immune Cells Via the TLR7 MyD88‐Dependent Signaling Pathway,” Nature Immunology 3, no. 2 (2002): 196–200. 11812998 10.1038/ni758

[96] R. L. Coffman , A. Sher , and R. A. Seder , “Vaccine Adjuvants: Putting Innate Immunity to Work,” Immunity 33, no. 4 (2010): 492–503. 21029960 10.1016/j.immuni.2010.10.002 PMC3420356

[97] Y. Choi , J. W. Bowman , and J. U. Jung , “Autophagy During Viral Infection ‐ A Double‐Edged Sword,” Nature Reviews Microbiology 16, no. 6 (2018): 341–354. 29556036 10.1038/s41579-018-0003-6 PMC6907743

[98] K. Cadwell , “Crosstalk Between Autophagy and Inflammatory Signalling Pathways: Balancing Defence and Homeostasis,” Nature Reviews Immunology 16, no. 11 (2016): 661–675. 10.1038/nri.2016.100 PMC5343289 27694913

[99] J. Zhang , W. Han , C. Xie , et al., “Autophagy Inhibitors Alleviate Japanese Encephalitis Virus‐Induced Cerebral Inflammation in Mice,” Archives of Virology 167, no. 3 (2022): 849–859. 35119507 10.1007/s00705-021-05283-9 PMC8814803

[100] F. Zhao , Y. Zhai , J. Zhu , P. Xiao , and G. Feng , “Enhancement of Autophagy as a Strategy for Development of New DNA Vaccine Candidates Against Japanese Encephalitis,” Vaccine 37, no. 37 (2019): 5588–5595. 31399273 10.1016/j.vaccine.2019.07.093

[101] L. Turtle and T. Solomon , “Japanese Encephalitis ‐ The Prospects for New Treatments,” Nature Reviews Neurology 14, no. 5 (2018): 298–313. 29697099 10.1038/nrneurol.2018.30

